# High-flow nasal cannula oxygen therapy: physiological basis and clinical applications in anesthesia

**DOI:** 10.3389/fmed.2025.1661569

**Published:** 2025-09-26

**Authors:** Hui Liu, Peng Qu, Qian Liu, Fengfeng Xiao, Yanling Yang, Liu Xu, Hongyan Zhang

**Affiliations:** ^1^Department of Anesthesiology, Chengdu Wenjiang District People's Hospital, Chengdu, China; ^2^School of Laboratory Medicine, North Sichuan Medical College, Nanchong, China; ^3^Institute of Cardiovascular Diseases & Department of Cardiology, Sichuan Provincial People's Hospital, School of Medicine, University of Electronic Science and Technology of China, Chengdu, China

**Keywords:** HFNC, physiological mechanisms, perioperative care, special populations, anesthesia practice

## Abstract

High-flow nasal cannula (HFNC) oxygen therapy, a non-invasive respiratory support modality, has gained increasing attention for its advantages in perioperative care. This review outlines the basic components and key physiological effects of HFNC, including apneic oxygenation, positive end-expiratory pressure (PEEP), reduction of anatomical dead space, enhanced end-expiratory lung volume, accurate oxygen delivery, and active humidification. These mechanisms support its application across multiple perioperative phases, such as tracheal intubation, sedation for endoscopic procedures, upper airway surgeries, extubation, and recovery. HFNC has also shown promise in specific patient populations, including the obese, pregnant, and pediatric patients. Although its clinical benefits and safety profile are well-recognized, further studies are needed to clarify its indications, refine device settings, and explore its integration with other respiratory strategies. This review aims to summarize current clinical applications and recent developments of HFNC in anesthesia practice, providing both theoretical context and practical recommendations for its standardized use.

## 1 Introduction

HFNC therapy has gained prominence as a contemporary, non-invasive respiratory-support modality and is now embedded in routine critical-care and peri-operative practice. By delivering warmed, fully humidified gas at flow rates of 40–80 L min^−1^, HFNC maintains a constant, titratable fraction of inspired oxygen (FiO_2_) while generating a modest level of positive end-expiratory pressure (PEEP) (1, 2). In addition, the continuous flow flushes nasopharyngeal dead space, reduces work of breathing, and supports apnoeic oxygenation—the passive diffusion of oxygen in an apnoeic but patent airway—an effect of direct relevance to anesthetic induction and airway management (3–5). Compared with conventional low-flow oxygen interfaces such as standard nasal prongs and face masks, HFNC provides more reliable oxygen delivery, greater patient comfort, superior humidification, and an extended safe-apnoea window (6, 7). As a result, it has supplanted conventional oxygen therapy (COT) in many intensive-care settings, particularly for acute hypoxaemic respiratory failure, and is now advocated as first-line support in numerous guidelines (8–10). Peri-operatively, interest in HFNC has broadened rapidly. Reported benefits span the full anesthetic continuum: pre-oxygenation and induction of general anesthesia, management of the anticipated or unanticipated difficult airway, oxygenation during non-invasive endoscopic or airway procedures, facilitation of tubeless microlaryngeal surgery, support during emergence and extubation, and mitigation of early postoperative respiratory failure. Transnasal humidified rapid-insufflation ventilatory exchange (THRIVE) refers to a specific clinical application of HFNC in which 100% oxygen is delivered at very high flow rates (often up to 90 L·min^−1^) to maximize apnoeic oxygenation during airway management. Although utilizing the same technology as HFNC, the term “THRIVE” emphasizes its strategic use in extending safe apnoea time and maintaining an unobstructed surgical field during upper-airway interventions (11, 12). Despite these advances, robust, standardized guidance for anesthetists on patient selection, FiO_2_/flow titration, monitoring of carbon-dioxide accumulation, and criteria for escalation or weaning remains limited. Practice therefore varies widely between centers. This narrative review (i) outlines the physiological mechanisms that underpin HFNC efficacy; (ii) collates and appraises contemporary evidence across core anesthetic milestones; and (iii) examines emerging data in special populations—including obese, obstetric, pediatric, and cardiac-surgical patients—where oxygen reserves are precarious and airway management challenging. By integrating mechanistic insight with clinical trial data, we aim to provide anesthesiologists with pragmatic, evidence-based recommendations to optimize peri-operative respiratory care and standardize HFNC use across diverse practice environments.

## 2 Structure and physiological effects of HFNC

### 2.1 Structural overview of HFNC

**Figure 1:**
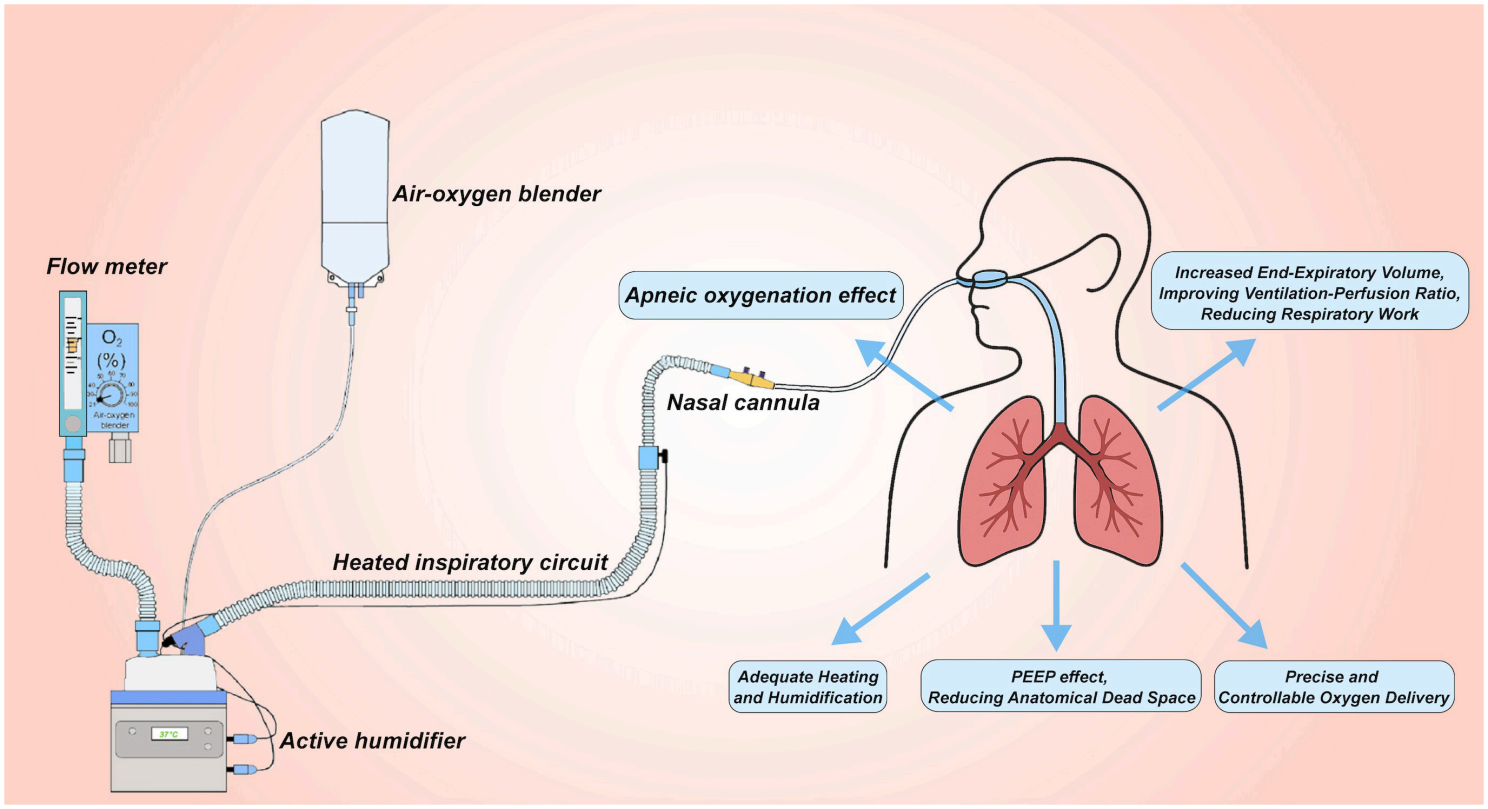
Setup for high-flow nasal cannula (HFNC) oxygen therapy. The system uses an air-oxygen blender to deliver controlled oxygen concentrations (FiO2) from 0.21 to 1.0 at up to 60 L/min flow. The gas is heated and humidified through an active humidifier, delivered via a heated inspiratory circuit, and administered through large-diameter nasal cannulas. This setup provides several benefits: (1) no-breathing oxygenation effect; (2) PEEP Effect, reducing anatomical dead space; (3) increased End-Expiratory Volume, improving ventilation-perfusion ratio and reducing respiratory work; (4) precise and controllable oxygen delivery; (5) adequate heating and humidification to optimize patient comfort and respiratory function.

A standard HFNC set-up integrates four functional modules: (i) an air–oxygen blending unit, (ii) a heated humidifier, (iii) delivery circuits, and (iv) the nasal interface (13, 14). Air–oxygen blender. Ambient air and pressurized oxygen are mixed to the desired fraction of inspired oxygen before entering the turbine. FiO_2_ may be adjusted in two ways. The traditional float-type flowmeter alters oxygen flow and achieves an estimated FiO_2_ but lacks precise preset capability. Modern systems incorporate a micro-proportional valve coupled to an ultrasonic oxygen sensor, allowing programmable, closed-loop FiO_2_ control. Heated humidifier. The blended gas is conditioned to 31–37°C and fully saturated, protecting mucosal integrity and improving comfort. Gas-delivery circuit and cannula. Heated, humidified gas (8–80 L min^−1^, FiO_2_ 0.21–1.00) is conveyed through insulated tubing to a soft, beveled nasal cannula secured with an adjustable head strap (14, 15). This configuration delivers a small level of positive end-expiratory pressure, maintains stable oxygen concentration, and enhances patient tolerance (Figure 1). Together, these components provide a high-flow, temperature- and humidity-controlled oxygen source that underpins the clinical advantages of HFNC therapy.

### 2.2 The physiological effects of HFNC

#### 2.2.1 Oxygenation effect without respiratory effort

HFNC therapy supplies a heated, humidified, high-flow oxygen mixture via a bespoke nasal interface, generating a modest but continuous positive airway pressure. The resulting alveolar–capillary oxygen gradient supports passive diffusion and sustains arterial oxygenation even in the absence of ventilatory effort (3). By prolonging the “safe apnoea” window—defined as the interval during which peripheral oxygen saturation (SpO_2_) remains ≥90% without ventilation (16)—HFNC has become an important adjunct during rapid-sequence induction, anticipated difficult airway management, and emergency intubation (4, 17). Patel et al. (18) applied THRIVE, a high-flow variant, in 25 patients with challenging airways undergoing oropharyngeal or laryngeal surgery. Median safe apnoea time reached 14 min (interquartile range, 9–19 min), illustrating HFNC's ability to maintain oxygenation while clinicians secure the airway.

#### 2.2.2 PEEP effect and reduction of anatomical dead space

PEEP is the residual pressure maintained in the airway at end-expiration during mechanical ventilation. By preventing alveolar collapse, PEEP promotes recruitment of previously nonaerated lung units, thereby enhancing oxygenation and overall pulmonary mechanics (19, 20). Appropriately titrated PEEP helps counter atelectasis and hypoxaemia throughout anesthetic induction, intra-operative ventilation, and postoperative respiratory care (21–23). HFNC therapy produces a comparable pressure effect. In healthy volunteers, closed-mouth breathing with HFNC flow rates of 10, 20, 40, and 60 L min^−1^ generated mean airway pressures of 1.7, 2.9, 5.5, and 7.4 cm H_2_O, respectively—a near-linear flow–pressure relationship; opening the mouth lowered each value by roughly 1 cm H_2_O (24). Continuous high-flow insufflation also flushes the nasopharyngeal dead space, expelling retained CO_2_, limiting rebreathing, and raising the effective fraction of inspired oxygen. These combined effects improve alveolar ventilation and gas exchange, with efficacy proportional to both flow magnitude and duration of use (3, 25, 26).

#### 2.2.3 Increase in end-expiratory volume, improvement of ventilation-perfusion ratio, and reduction of work of breathing (WOB)

WOB denotes the metabolic energy consumed by the respiratory muscles. Measures that lower breathing frequency and enhance thoraco-abdominal synchrony help curb this demand. The modest positive end-expiratory pressure generated by HFNC offsets intrinsic PEEP and recruits atelectatic units, thereby unloading inspiratory effort (27, 28). Concurrently, HFNC enlarges end-expiratory lung volume (EELV) and global lung impedance, the latter is used as a surrogate for end-expiratory lung volume (EELV), in mechanically ventilated patients, stepwise PEEP increase causes proportional increases in both EELV and end-expiratory lung impedance, with a demonstrated high linear correlation (R^2^ ≈ 0.95) (29). Thus, lung impedance provides a reliable bedside indicator of changes in lung volume (30). Electrical-impedance tomography confirmed flow-dependent rises in EELV in both supine and prone positions; the increase was evenly distributed prone but localized to dependent (ventral) zones supine (31). By recruiting alveoli, smoothing regional ventilation, and reducing inspiratory effort, HFNC enhances ventilation–perfusion matching. Compared with non-invasive ventilation or conventional oxygen therapy, it yields greater tidal volumes, higher dynamic compliance, and more complete re-expansion of collapsed lung (32, 33), while reducing airway resistance and inspiratory workload (32, 34). In a crossover study of 22 recently extubated ICU patients, 1 h of prophylactic HFNC lessened respiratory load, increased EELV, and improved PaO_2_/FiO_2_ vs. standard oxygen, without affecting hemodynamics or cardiac biomarkers (35). Collectively, these findings highlight HFNC's capacity to meaningfully reduce WOB and optimize gas exchange during the peri-extubation period.

#### 2.2.4 Controlled and precise oxygen delivery

HFNC systems can provide up to 70–80 L min^−1^ of warmed, humidified gas, while an integrated air–oxygen blender allows fraction of inspired oxygen adjustment from 0.21 to 1.00, independent of flow setting (36). To avoid dilution by entrained room air, the delivered flow must at least match the patient's peak inspiratory demand (37). FiO_2_ accuracy also hinges on oral closure: nasal breathing with the mouth shut minimizes ambient mixing and preserves the prescribed concentration, whereas mouth opening lowers the effective FiO_2_ (38).

#### 2.2.5 Adequate heating and humidification

Conventional oxygen devices delivering flows ≥ 6 L min^−1^ often desiccate and irritate the nasal mucosa, provoking discomfort and poor tolerance. Even brief exposure to inadequately humidified gas has been shown to impair airway-epithelial function (39). Optimal ciliary activity, which facilitates mucociliary clearance, requires an inspired-gas temperature of approximately 37°C (40). Contemporary HFNC systems incorporate active heating and humidification, supplying gas at up to 37°C with an absolute humidity of 44 mg H_2_O L^−1^ (100 % relative humidity). This conditioning maintains secretion mobility and supports efficient ciliary transport (41, 42), a particular advantage in patients with copious airway secretions where effective clearance is crucial (see Figure 1).

## 3 Clinical applications of HFNC in anesthesia

HFNC therapy is now used across a spectrum of peri-operative scenarios, including management of acute hypoxaemic respiratory failure, post-extubation support, pre-oxygenation before tracheal intubation or bronchoscopy, rescue of early postoperative respiratory compromise, and the intrahospital transfer of critically ill patients (43–45). HFNC is unsuitable when upper-airway obstruction or severe anatomical distortion precludes effective flow delivery, or in situations of life-threatening hypoxaemia, marked haemodynamic instability, facial or skull-base trauma, and untreated pneumothorax. Caution is also advised in patients with depressed consciousness, congenital cardiac shunts, acute bronchospasm, or chronic hypercapnic respiratory failure (14, 46, 47).

### 3.1 HFNC in tracheal intubation

Tracheal intubation is ubiquitous in anesthetic practice, yet the apnoea that accompanies induction eliminates spontaneous ventilation and jeopardizes continuous oxygen delivery. The danger is amplified when airway management proves difficult, as prolonged intubation attempts markedly heighten the risk of hypoxaemia. Although pre-oxygenation is routinely employed to buffer oxygen saturation (SpO_2_) during laryngoscopy, its efficacy is variable, and critically ill patients often fail to reach optimal end-tidal oxygen levels (≈ 90 %) with traditional techniques (48). HFNC therapy—capable of maintaining oxygenation throughout the apnoeic interval—therefore offers a compelling alternative to conventional pre-oxygenation strategies.

#### 3.1.1 HFNC in preoxygenation

HFNC therapy delivers a continuous, heated, humidified oxygen stream at high flow, permitting apnoeic oxygenation as long as the airway remains patent. The sustained oxygen–carbon dioxide diffusion gradient allows alveolar O_2_ uptake to exceed the rate of progressive CO_2_ retention, thereby extending the “safe-apnoea” window and supporting oxygenation during intubation attempts (49). Traditional pre-oxygenation relies on a well-sealed face mask (FM) attached to the breathing circuit (flow > 6 L min^−1^) with either 3 min of quiet breathing or ≥ 4 vital-capacity breaths (50). Mask leak or poor tolerance, however, can undermine effectiveness and heighten the risk of peri-induction hypoxaemia. HFNC circumvents these limitations by supplying high-flow oxygen through nasal prongs, independent of mask seal. A meta-analysis of 14 randomized controlled trials (*n* = 1,012) showed that HFNC pre-oxygenation improved arterial PaO_2_ (mean difference ≈ 57 mm Hg) and lengthened safe-apnoea time (≈87 s) relative to FM, without altering minimum SpO_2_, PaCO_2_/EtCO_2_, end-tidal oxygen, or desaturation slope (51). In emergency intubations, 3 min of THRIVE (60 L min^−1^) similarly lowered hypoxaemia incidence in high-risk patients (52). Collectively, these data support using HFNC both for pre-oxygenation and continuous oxygen delivery throughout laryngoscopy, especially in anticipated difficult airways, thereby enhancing intubation safety (53).

#### 3.1.2 HFNC in difficult airway management

Maintaining spontaneous ventilation during fiber-optic or video-assisted laryngoscopy is central to the management of anticipated difficult airways (42). A HFNC can deliver continuous, heated, humidified oxygen through the nares without obstructing the oral route, thereby sustaining arterial oxygenation while the bronchoscope or tracheal tube is advanced. This uninterrupted flow optimizes intubation conditions and may mitigate peri-procedural hypoxaemia. In 2015, Patel and Nouraei applied THRIVE in a cohort of high-risk difficult airway patients, achieving a median apnoea time of 14 min after induction without ventilation and without any occurrence of SpO_2_ < 90%, thereby establishing its physiological and clinical feasibility in this setting (18). Subsequently, a systematic review of 14 randomized controlled trials (*n* = 1,012) demonstrated that, compared with conventional facemask oxygenation, HFNC during anesthetic induction significantly increased PaO_2_ and prolonged the safe apnoea time by ~87 s (51). In intraoperative scenarios involving anticipated difficult airways, the PREOPTI-DAM randomized trial found no statistically significant difference between HFNC (4-min preoxygenation) and facemask oxygenation in the primary composite endpoint (SpO_2_ ≤ 94% or the need for facemask ventilation); however, patients reported better comfort, suggesting HFNC is at least non-inferior to facemask oxygenation while providing the added convenience of continuous oxygen delivery throughout the intubation process (54). In critically ill patients undergoing preoxygenation and intubation, multicentre ICU RCTs and meta-analyses have shown that HFNC may reduce the incidence of intubation-related adverse events and severe hypoxaemia, although improvements in nadir SpO_2_ vary depending on the patient population and baseline oxygenation status; combining HFNC with NIV may further enhance efficacy (55). For awake tracheal intubation (ATI), the main advantages of HFNC are its ability to maintain oxygenation without interrupting airway access: it does not obstruct oral or nasal routes, allowing adequate topical anesthesia and manipulation of fiber-optic bronchoscopes or video laryngoscopes while sustaining high SpO_2_ levels throughout the procedure (56, 57). Both retrospective and prospective studies have demonstrated that, compared with low-flow oxygen, HFNC during ATI improves the lowest SpO_2_ achieved and reduces the need for multiple intubation attempts. The 2022 ASA Difficult Airway Guidelines explicitly recommend continuous oxygen delivery—including via nasal routes—before, during, and after intubation, supporting its use in ATI (58). Practical recommendations include administering FiO_2_ 1.0 at a flow rate ≥40–60 L/min for at least 3–4 min before induction, maintaining HFNC until the tracheal tube is secured, keeping the mouth closed and elevating the mandible to optimize pharyngeal pressure, and initiating awake-phase HFNC at 20–40 L/min for improved tolerance with subsequent titration as required. It should be noted that HFNC primarily addresses hypoxaemia but is ineffective for CO_2_ clearance; prolonged apnoea may lead to hypercapnia and acidosis. Therefore, the duration of apnoea should be limited, ventilation should be closely monitored, and the combination with NIV or other ventilation strategies should be considered on an individual basis (14).

#### 3.1.3 HFNC in rapid sequence induction (RSI) for high-risk patients

RSI is often selected for patients with a full stomach, gastrointestinal obstruction, or other aspiration risks to minimize the interval between loss of protective reflexes and tracheal intubation. RSI aims to reduce aspiration risk by minimizing the time between loss of airway reflexes and securing the airway via tracheal tube placement (59). When applied during both pre-oxygenation and the subsequent apnoeic phase, HFNC therapy can enhance arterial oxygenation and lengthen the safe-apnoea window, although continuous flow may contribute to gastric insufflation and, theoretically, reflux. In older adults (≥65 yr) undergoing emergency laparotomy for bowel obstruction, pre-oxygenation with transnasal humidified rapid-insufflation ventilatory exchange (THRIVE: FiO_2_ 1.0, 40 L min^−1^, closed mouth, 5 min) raised PaO_2_ to 446 ± 84 mm Hg vs. 262 ± 31 mm Hg with face-mask (FM) pre-oxygenation (FiO_2_ 1.0, 6 L min^−1^) and doubled median safe-apnoea time (480 vs. 240 s) (60). Similar findings were reported by Mir et al. in 40 emergency surgical patients: THRIVE extended apnoea duration (248 ± 71 vs. 123 ± 55 s) without altering PaO_2_, PaCO_2_ or pH, and no participant in either group desaturated below SpO_2_ 90% (61). In a prospective, unblinded randomized trial, minimum SpO_2_ 1 min post-intubation was comparable between THRIVE and FM cohorts; however, 18% of FM patients fell below SpO_2_ 96%, whereas all THRIVE recipients remained ≥ 96% (62).

In conclusion, HFNC can serve as an effective preoxygenation method before tracheal intubation and provide continuous oxygenation support during the intubation process, particularly in patients with difficult airways. It demonstrates significant advantages in prolonging the safe apnea time, optimizing intubation conditions, and reducing the incidence of hypoxemia.

### 3.2 Application of HFNC in painless endoscopic procedures

**Figure 2:**
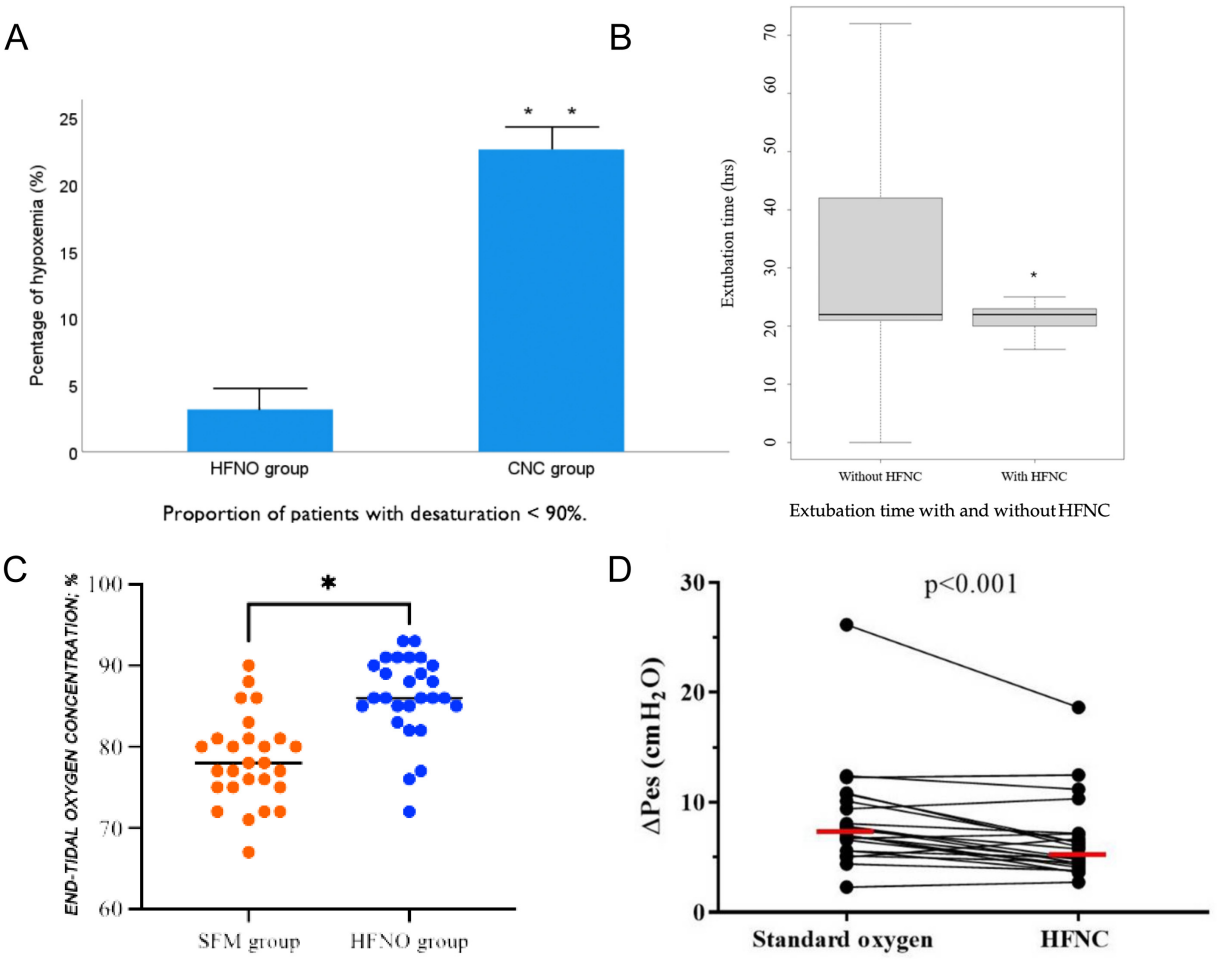
**(A)** HFNO significantly reduces the risk of post-extubation hypoxemia, especially in patients sensitive to oxygen levels (66). **(B)** HFNO significantly shortens extubation time and results in more consistent and stable extubation durations (75). **(C)** HFNO significantly increases end-tidal oxygen concentration (EtO_2_), reducing the risk of hypoxemia in high-risk parturients (90). **(D)** HFNC oxygen therapy after extubation reduces the work of breathing, suggesting advantages in lowering metabolic load and respiratory effort. The horizontal red line represents the median (35). *Indicates statistical significance (*p < 0.05, **p < 0.01).

Sedation, partial airway obstruction and suppression of respiratory drive predispose patients to peri-procedural hypoxaemia, with oxygen saturation frequently falling below 90% despite supplemental oxygen (63, 64). Non-invasive ventilation can mitigate this risk, yet poor tolerance of a tight face-mask and technical difficulty introducing the endoscope limit its routine use (65). By contrast, HFNC therapy leaves the oral route unobstructed, generates low-level positive end-expiratory pressure and improves alveolar ventilation (25), making it an attractive alternative during painless endoscopy. In elderly patients (≥65 yr) undergoing sedated gastroscopy, HFNC set at 60 L min^−1^ (FiO_2_ 0.6) reduced hypoxaemia from 22.6 to 3.2% compared with a conventional nasal cannula delivering 6 L min^−1^ (Figure 2A) (66). A larger, multicentre trial of nearly 2 000 ASA I–II patients confirmed these findings: HFNC (30–60 L min^−1^) abolished moderate and severe desaturation events that occurred with low-flow oxygen (67). A meta-analysis encompassing 2,998 subjects likewise demonstrated consistent reductions in hypoxaemia, severe desaturation and procedure interruptions across all endoscopy types and age groups (68). Evidence in bronchoscopy mirrors these benefits. During broncho-alveolar lavage, HFNC maintained PaO_2_, preserved end-expiratory lung impedance and lowered hypoxaemic episodes (11 vs. 56%) relative to standard oxygen therapy in a randomized study of out-patients (69). In a larger trial of 600 patients with chronic obstructive pulmonary disease, high-flow oxygen (60–80 L min^−1^, FiO_2_ 0.6–0.8) halved both cumulative hypoxaemia time and the number of desaturation events vs. low-flow therapy, without compromising comfort (odds ratio 5.1; 95 % CI 3.2–8.2; *p* < 0.001) (70). Collectively, these studies highlight the substantial potential of HFNC in painless endoscopic procedures. However, several challenges remain. Firstly, there is currently no standardized recommendation for optimal flow settings in different procedural contexts. Secondly, large-scale, multicenter trials are needed to further validate the long-term clinical benefits of HFNC. With continued technological advancement and accumulating evidence, HFNC is poised to become a standardized oxygenation modality in painless endoscopy.

### 3.3 Application of HFNC in patients undergoing laryngeal surgery

The role of HFNC therapy during laryngeal procedures is under active evaluation. In cases that require a shared airway—such as vocal-cord biopsy, tracheoscopy and dilation of subglottic stenosis—HFNC may obviate the need for tracheal intubation or jet ventilation and extend the apnoeic oxygenation window (71, 72). In 19 ASA I–II adults (body-mass index < 30 kg m^−2^) undergoing microlaryngoscopic resection of vocal-cord lesions, HFNC alone (60 L min^−1^; FiO_2_ 0.95) provided a mean apnoea time of 21.5 min (maximum 35 min). Peripheral oxygen saturation remained ≥ 90% in 18 patients; one transiently fell to 88%. Arterial carbon-dioxide tension rose linearly (mean 1.68 ± 0.12 mm Hg min^−1^) to a peak of 79.4 mmHg (73). A separate randomized study of ASA I–II patients undergoing short laryngeal procedures used transnasal humidified rapid-insufflation ventilatory exchange (THRIVE: 100% O_2_, 40–70 L min^−1^). Mean safe apnoea time was 22.5 min, with no patient desaturating below 91%. PaCO_2_ and end-tidal CO_2_ increased by 0.24 and 0.12 kPa min^−1^, respectively—slower than the rise expected during conventional apnoea (71). These findings suggest that, in non-obese patients without major co-morbidities, THRIVE can maintain oxygenation for up to 30 min during laryngeal surgery. Nevertheless, vigilant monitoring of PaCO_2_ and arterial pH is advisable to mitigate hypercapnia and acidaemia.

### 3.4 Application of HFNC in extubation

Post-extubation complications such as airway obstruction, respiratory distress, and hypoxemia are associated with increased pulmonary infection risk and prolonged hospitalization (74). HFNC therapy has been shown to reduce the need for reintubation in both postoperative and medically complex patient cohorts. HFNC offers continuous oxygen delivery, increases end-expiratory lung volume, improves alveolar ventilation and lung compliance, and promotes re-expansion of collapsed alveoli. These physiological effects optimize ventilation–perfusion matching and enhance oxygenation (25). Additionally, active warming and humidification preserve mucosal integrity and facilitate secretion clearance by enhancing ciliary function. In patients with obstructive sleep apnea (OSA), HFNC—with or without an airway exchange catheter (AEC)—was associated with shorter time to extubation and fewer hypoxemic episodes, without major adverse events (75) (Figure 2B). A multicenter randomized controlled trial comparing HFNC (50 L·min^−1^, FiO_2_ 0.5) with bilevel positive airway pressure (BiPAP: pressure support 8 cm H_2_O, PEEP 4 cm H_2_O, FiO_2_ 0.5, ≥4 h/day) in 830 patients with acute respiratory failure after cardiothoracic surgery found no significant differences in treatment failure or ICU mortality between groups, supporting HFNC as a non-inferior and better-tolerated alternative (Figure 2D) (76). A meta-analysis of 11 RCTs involving 2,201 patients further demonstrated that HFNC initiated immediately postoperatively significantly reduced reintubation rates and the need for escalation of respiratory support compared to COT, particularly in high-risk and obese patients (77). Another RCT showed that early application of HFNC after extubation led to better oxygenation, improved patient comfort, and fewer pressure-related mucosal injuries compared to standard face mask oxygen therapy (78). Collectively, these findings support HFNC as a safe and effective post-extubation strategy. Its clinical benefits—comparable to those of non-invasive ventilation (NIV)—include reduced respiratory effort, improved oxygenation, and enhanced patient tolerance, particularly in high-risk populations such as those undergoing cardiothoracic surgery. In appropriately selected patients, HFNC may be considered a more comfortable and equally effective alternative to facemask NIV, although evidence regarding helmet NIV is still lacking.

## 4 Application of HFNC in anesthesia for special populations

### 4.1 Application of HFNC in anesthesia for obese patients

Obesity is a well-recognized risk factor for perioperative hypoxemia and difficult airway management, attributed to anatomical and physiological alterations such as excessive adipose deposition, diaphragmatic elevation, reduced lung compliance, and decreased functional residual capacity (79). In patients with morbid obesity—particularly those with obstructive sleep apnoea–hypopnoea syndrome (OSAHS)—NIV, including CPAP or BiPAP, is widely regarded as the most effective strategy for optimizing pre-oxygenation and prolonging safe apnoea time (80). Nevertheless, HFNC therapy offers rapid and sustained oxygen delivery, augments oxygen reserves, and extends the safe apnoea window, thereby lowering the risk of peri-intubation and intraoperative desaturation in selected patients, especially when NIV is not feasible or poorly tolerated (81). A 2022 meta-analysis by Zhou et al. included 12 randomized controlled trials (RCTs) involving 798 obese patients and found that, compared to COT, HFNC significantly reduced the incidence of hypoxemia, increased lowest SpO_2_ values, decreased the need for additional respiratory support, and shortened hospital stay (82). Another meta-analysis of six RCTs (*n* = 351) comparing HFNC and face-mask (FM) oxygenation during general anesthesia found no significant differences in the occurrence of pre-intubation SpO_2_ < 92% or minimum SpO_2_; however, HFNC notably prolonged the duration of safe apnoea (83). In a cohort of 25 obese patients (BMI > 30 kg m^−2^) with difficult airways undergoing upper airway surgery, the use of transnasal THRIVE extended the mean apnoea time to 14 min, offering clinicians additional time for airway intervention (18). A separate case report described the use of HFNC in a morbidly obese patient (BMI 90 kg m^−2^) with COVID-19 pneumonia and obstructive sleep apnea. The patient, classified as ASA IV-E, underwent emergency laparotomy after preoxygenation with HFNC, which facilitated successful induction and intubation; alternating HFNC and BiPAP in the ICU subsequently maintained SpO_2_ at 91–92% (FiO_2_ 0.6) (84). Further evidence from bariatric surgery populations demonstrates that HFNC enhances arterial oxygenation during preoxygenation. Compared to FM and continuous positive airway pressure (CPAP), HFNC achieved higher PaO_2_ values after 3 min of preoxygenation. Prolonging the preoxygenation period beyond 5 min did not result in further PaO_2_ gains (85).

In summary, HFNC provides distinct advantages in the perioperative care of obese patients—including improved pre-intubation oxygenation, reduced reintubation risk, extended apnoea time, and greater patient tolerance—supporting its role as a preferred oxygenation strategy in this high-risk population.

### 4.2 Application of HFNC in obstetric anesthesia

Pregnancy-associated reductions in functional residual capacity, coupled with heightened oxygen consumption, place parturients at particular risk of rapid desaturation and difficult airway management. During rapid-sequence induction (RSI), up to 17% of women experience SpO_2_ < 90% (86). Maternal hypoxaemia jeopardizes fetal oxygenation and may precipitate acidaemia, underscoring the need for prompt, reliable oxygen delivery throughout induction and intubation (87, 88). HFNC provides a continuous, heated, humidified oxygen stream that augments alveolar ventilation, improves lung compliance, and expands oxygen reserves during induction, laryngoscopy, and extubation. Its safety in pregnant patients with acute respiratory failure has been confirmed (89). In a prospective, dual-center trial of parturients (BMI > 30 kg m^−2^) undergoing cesarean delivery under general anesthesia, HFNC significantly increased oxygen reserve and extended safe-apnoea time, thereby reducing peri-intubation hypoxaemia (Figure 2C) (90). Comparative data further support these advantages. In term pregnancies, 3 min of HFNC at 70 L min^−1^ achieved an end-tidal oxygen concentration ≥ 90 % in 71 % of subjects vs. 44 % with face-mask (FM) oxygenation at 10 L min^−1^ (91). Similarly, HFNC during RSI for cesarean section improved immediate post-intubation PaO_2_ relative to FM, without affecting nadir SpO_2_ or neonatal outcomes (92). Collectively, these studies indicate that HFNC is a safe, effective, and aspiration-neutral technique for obstetric airway management, offering superior oxygenation and a longer margin of safety compared with conventional methods.

### 4.3 Application of HFNC in pediatric anesthesia

HFNC therapy has gained widespread use in children and neonates due to its ease of setup, superior comfort, and non-invasive delivery profile (93). Flow rates in pediatric HFNC are typically weight- and age-adjusted (94). Compared with adults, children have lower functional oxygen reserves and higher metabolic oxygen demands, placing them at greater risk of hypoxemia during sedation, airway instrumentation, or apnea. HFNC enhances oxygenation, improves ventilation efficiency, and extends safe apneic duration, making it increasingly valuable in pediatric anesthesia for difficult airway management, post-extubation oxygen therapy, and sedation procedures (4). In a single-center randomized controlled trial (RCT), 120 children (aged 2–7 years, 10–30 kg) undergoing outpatient oral surgery under deep sedation were randomized to receive either HFNC with propofol intravenous anesthesia (HFNC-IV) or laryngeal mask airway with propofol (LMA-IV) (95). HFNC was administered at 2 L· kg^−1^·min^−1^. Results showed comparable oxygenation (SpO_2_ > 97%) and ventilation (TcCO_2_ mean difference = −1.4 mmHg) between groups, with better surgical field visibility in the HFNC group and no significant differences in adverse event rates. This study supports HFNC as an effective alternative to LMA in pediatric sedation, offering adequate oxygenation and superior procedural access under conditions of spontaneous ventilation. Another RCT evaluated THRIVE in 48 healthy children (0–10 years) undergoing elective procedures under general anesthesia with neuromuscular blockade (96). HFNC flow rates were stratified by weight (5–15 kg: 2 L·kg^−1^·min^−1^; 15–30 kg: 35 L·min^−1^; 30–50 kg: 40 L·min^−1^; >50 kg: 50 L·min^−1^). Compared to controls, THRIVE significantly prolonged safe apnea time (by 80–170 s across weight groups) and maintained SpO_2_ at 99.6%, with no difference in CO_2_ accumulation rates (2.4 mmHg·min^−1^). These findings highlight THRIVE's ability to extend apneic tolerance in healthy pediatric patients, though it does not enhance CO_2_ clearance. In a separate study of preterm infants undergoing glottic stenosis dilation under general anesthesia, HFNC was shown to extend apneic time, providing a valuable oxygenation bridge during complex neonatal airway procedures (97). However, findings are not universally positive. A non-blinded, single-center RCT comparing HFNC with COT during pediatric upper gastrointestinal endoscopy reported no significant improvements in respiratory stability, including hypoxemia, hypercapnia, or apnea (98). Taken together, the available evidence suggests that HFNC and THRIVE offer safe and effective oxygenation support in pediatric and neonatal populations, especially in procedures requiring spontaneous ventilation or shared airway access. However, clinical benefits may vary by procedure type and patient risk profile, warranting further research in specific pediatric subgroups.

HFNC therapy is gaining traction as a non-invasive respiratory support strategy after pediatric cardiac procedures, where rapid desaturation and increased work of breathing are common. Testa et al. (99) observed that, within 48 h of extubation, HFNC did not alter PaCO_2_ relative to COT but significantly improved PaO_2_ and PaO_2_/FiO_2_ ratios. In ten infants studied by Itagaki and colleagues (median age 7 months), a flow of 2 L kg^−1^ min^−1^—though not 1 L kg^−1^ min^−1^—reduced respiratory rate, minute ventilation and thoraco-abdominal asynchrony, indicating lower inspiratory workload (100). During catheter-based interventions in 200 children with non-cyanotic congenital heart disease, weight-adjusted HFNC (2 or 35–50 L min^−1^; FiO_2_ 0.40) raised the minimum SpO_2_ and curtailed rescue ventilation needs compared with a 5 L min^−1^ face-mask; hypoxaemia occurred in 7.1% of controls and in none of the HFNC group, without extra gastric or haemodynamic complications (101). Post-extubation, Kumar et al. (102) reported superior oxygenation with HFNC vs. non-invasive ventilation, with similar re-intubation rates and CO_2_ clearance. More recently, early HFNC use after cardiopulmonary bypass was associated with less atelectasis and fewer early re-intubations (103). Collectively, these findings suggest HFNC can enhance oxygenation, reduce respiratory effort, and provide well-tolerated support in children with congenital heart disease, although careful haemodynamic monitoring remains prudent in complex cardiac physiology.

## 5 Contraindications to HFNC therapy

Although HFNC therapy offers multiple clinical advantages, its use must be carefully considered in specific scenarios due to established contraindications. Absolute contraindications include complete upper airway obstruction, basilar skull or nasal bone fractures, and patient refusal—primarily reflecting anatomical limitations or ethical concerns. Relative contraindications encompass poor patient cooperation, procedures involving electrosurgical or laser use in the airway, elevated aspiration risk, and significant carbon dioxide retention. In these cases, individualized risk-benefit assessments are essential. Its use is limited in the settings outlined below (Tables 1, 2).

**Table 1:** Absolute contraindications.

**Condition**	**Rationale**	**References**
Complete upper-airway obstruction	HFNC depends on a patent airway and spontaneous ventilation; anatomical or functional occlusion (e.g., laryngeal mass, critical tracheal stenosis) precludes effective gas flow.	(93, 104)
Basilar-skull or nasal-bone fracture	Nasal injury may compromise cannula placement and humidified flow; skull-base disruption increases the theoretical risk of intracranial infection.	(93)
Patient refusal	Informed refusal represents an ethical barrier to therapy initiation.	–

**Table 2:** Relative contraindications.

**Condition**	**Clinical consideration**	**References**
Non-compliance or intolerance	Adequate seal and patient cooperation are required; agitation or discomfort can lead to treatment failure.	(105)
ENT laser or electrocautery surgery	High-flow oxygen increases the risk of airway fire and thermal injury; HFNC should be paused or FiO_2_ minimized during energy delivery.	(106)
Impaired airway protection/high aspiration risk	Continuous flow may worsen swallowing coordination and promote aspiration in patients with poor reflexes.	(93)
Marked CO_2_ retention (pH < 7.25)	HFNC provides limited carbon-dioxide clearance in acute decompensated hypercapnia; non-invasive ventilation remains the first-line modality in this context.	(107–109)

These contraindications reflect the underlying physiology and safety profile of HFNC. Absolute contraindications involve fixed anatomical or ethical barriers, whereas relative contraindications require individual risk–benefit assessment, taking into account surgical environment, aspiration risk, and the device's capacity for CO_2_ elimination.

## 6 Conclusion and outlook

HFNC oxygen therapy, as an emerging non-invasive respiratory support modality, has gained widespread clinical application owing to its advantages in improving oxygenation, reducing the WOB, and enhancing patient tolerance. Its efficacy is particularly notable in the management of mild to moderate hypoxemic respiratory failure. In anesthetic practice, HFNC offers a promising oxygenation support strategy during critical perioperative stages, such as difficult airway management, non-invasive airway interventions, postoperative recovery, and anesthesia for special populations, demonstrating strong clinical potential. However, the standardized use of HFNC still faces several challenges. There remains a lack of consensus and robust evidence regarding its indications, optimal parameter settings, and management strategies for high-risk populations. In addition, device capabilities require further refinement, particularly in areas such as pressure monitoring, automatic regulation, and integration with other respiratory support systems. Future research should focus on large-scale, prospective, multicenter clinical trials to clarify the clinical value of HFNC in various intraoperative and postoperative scenarios. At the same time, technological innovation should be pursued to improve device intelligence and system integration. In conclusion, as a bridging technology between COT and NIV, HFNC holds significant promise for optimizing perioperative respiratory management and improving patient outcomes, with substantial value for future research and clinical application.
